# Exploring the state of health of electric vehicle batteries at end of use; hierarchical waste flow analysis to determine the recycling and reuse potential

**DOI:** 10.1007/s13243-024-00137-4

**Published:** 2024-01-26

**Authors:** Narjes Fallah, Colin Fitzpatrick

**Affiliations:** Department of Electronic & Computer Engineering, University of Limerick, Limerick, Ireland

**Keywords:** Hierarchical waste estimation, End-of-life batteries, Electric vehicle, Repurposing

## Abstract

With the increasing adoption of electric vehicles, their end-of-life management is a timely matter. This requires recognizing the upcoming volume of retired electric-vehicle-batteries to the waste stream. The projection is further useful if we have an estimation of the remaining value within them to categorize the recycling or repurposing potential to allow appropriate policy development and facility planning. This qualification assessment is neglected in the current literature. Neglecting the health status of retired batteries in estimating their residual value might end up over or underestimating their reuse and recycling potential. This study aims to provide a hierarchical battery waste estimation based on their health and age of disposal in Ireland. These two factors are the fundamental parameters in determining the feasibility of repurposing or recycling retired batteries. Identifying this information, we defined three reuse scenarios with different state-of-health limits. Results indicate almost 50%, 30%, and below 10% repurposing potential in the year 2050 when setting a repurposing threshold of above 80%, 85%, and 90%, respectively. The authors also highlight the effect of non-regional repurposing on the recycling potential.

**Supplementary Information:**

The online version contains supplementary material available at 10.1007/s13243-024-00137-4.

## Introduction

A sustainable mobility policy suggests moving toward electrified transportation with promising environmental benefits for the reduction of GHG emissions while in use. However, there is often less attention given to their sustainability in other stages of the life cycle including raw material extraction, manufacturing, end-of-life treatment, and final disposal [29]. Particularly, for manufacturing, the significant investment in materials and resources for the production of large battery packs is an environmental drawback of the mass penetration of Electric Vehicles (EVs) in the transportation system [2]. This is even more challenging for remanufacturing since batteries are not suitably designed for such process [5]. Robert Casper and Erik Sundin [5] further discuss that the future actors and infrastructures are unset, suggesting additional investments into knowledge and equipment are required. Indeed, at disposal time, the unscheduled End-of-Life (EOL) management of EVs entails risks of unsustainability when they are still in their infancy [6, 11]. These environmental disadvantages underline the indefinite resilience and sustainability of a transition to fully electrified transportation and emphasise the requirement for circular economy strategies [4].

From a life cycle assessment perspective, EVs are more challenging to manage at EOL compared to traditional vehicles due to the inconsistency of the battery packages with other components in EVs in terms of lifetime performance and recycling treatments [11]. Although their life cycles are intertwined, the useful lifetime of the vehicle and the battery might not necessarily be the same as each other. Electric Vehicle Batteries (EVBs) are primarily designed for high acceleration applications in vehicles with great power-to-energy ratios, while in retirement, they might still meet the specific energy requirements of other applications [23]. Cascading the life cycle of EVBs by taking advantage of the retained capacity in a repurposed application could potentially reduce the net energy demand and carbon footprint and improve resource efficiency [22].

Recent investigations revealed that retired automobile batteries have the potential to be reused in several stationary applications, although some uncertainties remain in technical and economic terms [16]. The doubts surrounding the second-use viability of retired EVBs are partly due to the unclear market alignment and lack of financial justification along with technical uncertainties on safety and second-lifetime of batteries [4, 16], while the economic viability is not independent of the technical feasibility itself. In other words, the amount of energy that retired EVBs could offer to a second-use application depends on their State of Health (SOH) at the time of retirement and is crucial in the second-use viability assessment [21]. This underpins the importance of SOH categorization in the EOL EVB stream for second-life market assessment.

In the present literature, the investigations of battery repurposing are mostly assumption-based when it comes to SOH considerations [18, 20] (W. [27, 28]. These assumptions mainly follow the typical EOL limit of 70–80% of initial capacity (known as the knee point) for retired EVBs [12, 26] at which the batteries are assumed to have adequate energy capacity to contribute to a second life. In contrast to this, [17] demonstrate that for batteries to work in a long-lasting second application, they still need to be in their early degradation stage before reaching the knee point in the degradation curve. They argue that the 70 to 80% of remaining capacity in batteries at the time of retirement in the automobile is not a valid assumption as, in early retired EVs, batteries could still be at the slow stage of degradation. Other experimental studies on battery degradation [15] show a highly accelerated degradation stage started after the knee point suggesting that if batteries are retired during the slow degradation stage, the technical feasibility of second life is more sensible. However, the likelihood of early-degrading-stage retirement of modern automobile batteries with increased energy content and performance of Li-ion Batteries (LIBs) [14] needs further investigation.

While the early retirement of EVBs will increase their chance of being reused, this has not been accounted for in studies to estimate the EVB waste flow either. Generally, studies on the battery waste flow analysis take aggregated estimation without being specific about the age of retirement and SOH. For instance, [3] estimated the reuse capacity of retired EVBs by taking various collection rates, EOL remained capacity and reuse rates in the US. Their assumption on the health of batteries at EOL is averaged to 80%, 72%, and 64% of the initial capacity for the whole population, without any further investigation on age or health indication at disposal. Studies such as [13] and (Y. [27, 28] show a material composition in EVB waste without any specifications about reuse potential. Although [1] evaluated the impact of battery second use on the European EVB waste stream, their analysis takes a static second-life survival rate and without any SOH accountability in the evaluation. Moreover, in our previous work [10], the reuse capacity and volume are estimated by taking assumptions on the growing reuse rate and the SOH at EOL without further investigation on SOH assessment. To have a better understating of reuse potential in the retired battery stock and not rely on averaged rates, their health status needs to be represented in the waste flow analysis.

With this recognized gap in knowledge of the qualification of the EOL EVBs for reuse feasibility in the waste stream, the authors discovered that for such an assessment, one must consider that EVBs are disposed of across a distribution of ages and remaining SOH; this suggests a hierarchical EVB waste representation in various age and health class is crucial. Distinguishing these two fundamental parameters in retired EV batteries indicates that where these batteries are standing on the battery lifetime curve, consequently showing the residual value within them. When excluding these details from battery waste estimation, we might end up over or underestimating the recycling or repurposing potential. For instance, if we align recycling potential to the number of EV batteries at end-of-life and ignore or underestimate the repurposing potential, we end up oversizing the recycling supply. Especially when recycling facilities are competing with repurposing start-ups, knowing their secondary supply will help these businesses to properly size and scale. The knowledge of the qualified battery waste is also required to be regulated by the correspondent party for environmental considerations.

The authors in this work try to address this importance by including information on age and SOH at disposal of the EOL EVBs in their waste flow analysis. Furthermore, by setting different reuse thresholds for the health of EOL EVBs in three reuse scenarios, the reuse and recycling potentials are estimated. In this baseline, the delayed recycling of the reused EVBs is also considered by estimating the second-life survival rate of the repurposed EVBs with different SOH and ages of disposal.

The significance of addressing this research question is for EOL planning and management on a macro level. Recognizing the distribution of SOH of EOL EVBs disposed of across different ages would primarily assist waste policymakers in creating the required regulations for both recycling and reuse based on the reuse and recycling potential, and secondly supplement other second-life side businesses what to expect from the EVB waste on a big scale. It also matters greatly to those challenged with provisioning recycling facilities as there is a danger that assumptions that every EV will produce a battery for recycling upon retirement as a vehicle. Likewise, it must also be acknowledged that there may be a significant trans frontier shipment of used EVBs for repurposing which further complicates the planning of recycling capacity within primary markets.

The rest of the paper is as follows:

The next section describes the developed approach in this work including mathematical modelling. Findings are discussed in the result and discussion section followed by an overall conclusion on the essence of this work in the final section.

## Methodology

**Fig. 1:**
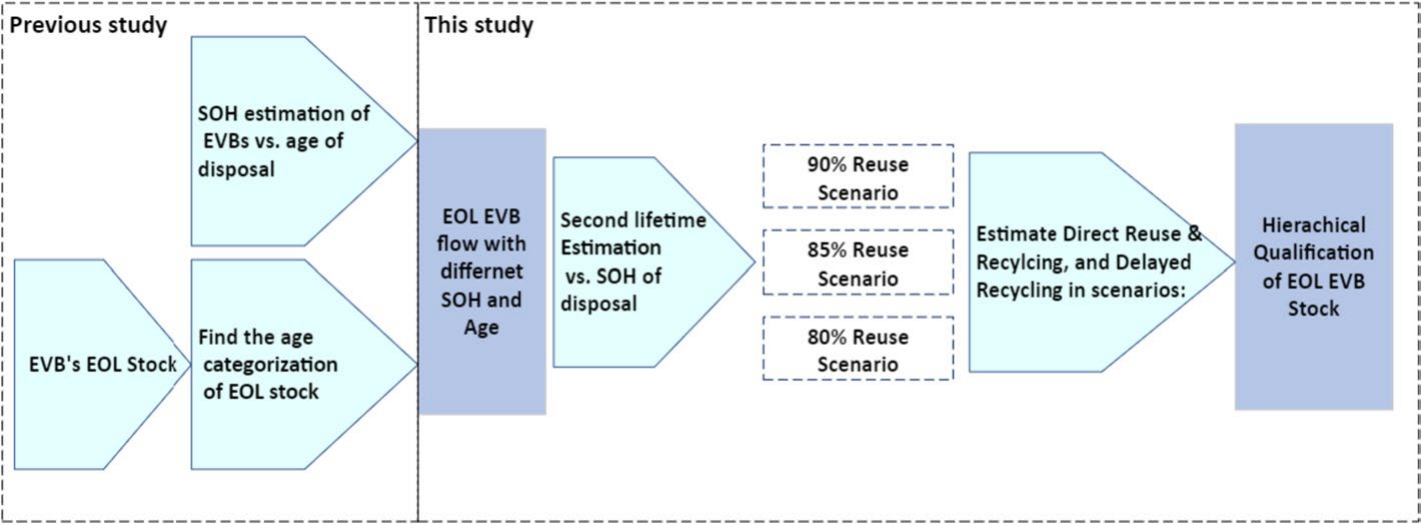
Methodology diagram

The goal of this study is approached through the step-by-step diagram represented in Fig. 1. This figure shows that the methodology in this work partly relies on our previous work as a baseline, whereas the rest is conducted for the qualification assessment of battery waste.

The target is to qualify the volume of the EOL EVB waste for recycling and reuse potential in Ireland. For this, an estimation of the number of units of EOL EVBs in the future waste stream is required. This has been accomplished for Ireland in our previous work [10] in which the unit of EOL battery packs are estimated using a material flow analysis and in benchmarks of nine scenarios defined based on various levels of the strictness of transport regulations and market potential. The analysis shows how many EVB units will reach EOL over the lifespan of EVs starting from their registration year. The results of the previous investigation are shown in aggregated numbers as figures in page 8 of [10] show, whereas in this study we also capture the age categorization of disposal as Eq. 1 suggests. This equation shows that the calculation of EOL units for every age of disposal $n$ is counted by multiplying the units sold in year $t$ to the probability of them reaching to EOL at the age of $n$.

1 $$\begin{array}{c}{EOL\_EVB}_n={Battery\_Units}_t\ast PDF.dist(n)\\\left\{\begin{array}{c}{EOL\_EVB}_n:number\;of\;EOL\;battery\;for\;every\;age\\n:age\;of\;disposal\;of\;EVs\\t:registration\;year\;of\;EVs\\PDF:probability\;distribution\;function\\dist:distribution\end{array}\right.\end{array}$$

In this work, the number of battery units (to be inserted in Eq. 1) within the registered EVs in the moderate scenario—2.B in page 8 in [10]—is considered for this evaluation to take a balanced perspective. This scenario assumes a medium level of regulation changes toward accepting EVs as well as moderate market growth and EV model availability in the market. Although, we took a step further in being conservative in approach by choosing a shorter spectrum of survival age of EVs in this work. In our previous work, the assumption was that EVs will go through an evolutionary change of peak survival age up to 17 years old due to the future technology and market growth during the next three decades, whereas in this study we take a range of 10 to12-year-old to be achieved by 2030. The reason for this assumption is that the 17 years peak survival is an optimistic assumption to be accomplished in this period. This data is available in the supplementary document. Note that efficiency improvement is neglected in this study, and this evaluation takes the current battery technology into analysis. Thus, this projection is more realistic for battery waste up for the next two decades since EVBs reaching EOL before 2040 are mostly the ones that have been sold before 2030, by which time the technology is not likely to evolve considerably.

**Fig. 2:**
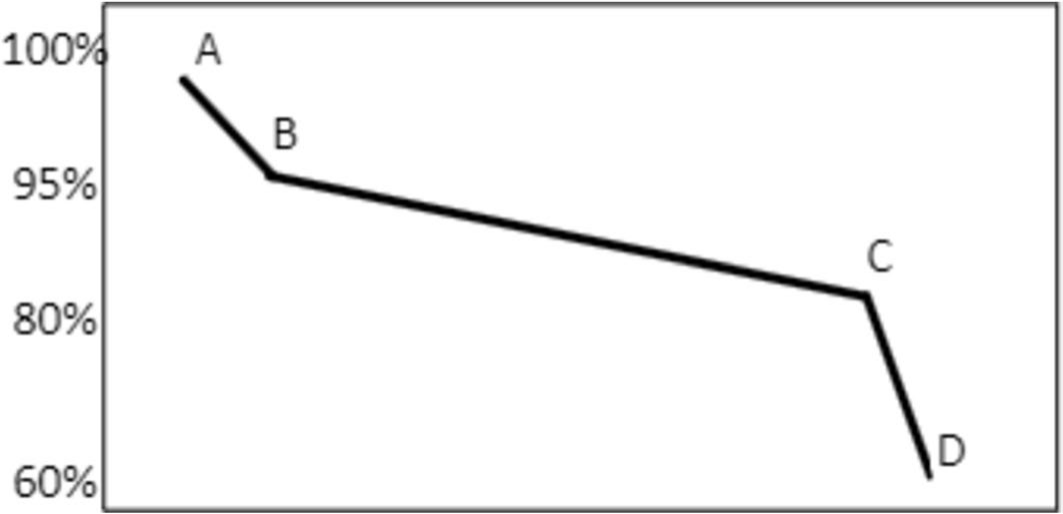
Capacity degradation profile of Li-ion battery-general figure [30]

Following the above analysis, the age categorization of EOL EVB waste is captured. Yet the age categorization without knowing their SOH cannot quite capture their status for repurposing or recycling potential. In fact, the age and the SOH at the time of retirement are the two critical factors for retired EVBs’ fate in the EOL stream since they define where they are positioned on the lifetime curve of battery cells. The age of disposal is important because the calendar life of battery cells is a logarithmic function and causes considerable degradation early in the battery use while over time it flattens out. The SOH at retirement shows how many more cycles batteries can deliver before reaching the EOL capacity threshold defined by OEMs. However, the calendar and cycling age estimation are blended in the remaining lifetime calculations and cannot be independently counted. The general behaviour of battery degradation [30] is represented in Fig. 2, wherein X axis shows the general representation of the number of deliverable cycles (The real values depends on the discharging profile i.e., depth of discharge and state of charge, thus is not a unique profile), and Y axis shows battery capacity. This profile shows that batteries degrade the first five per cent of their capacity at the very beginning (point A to point B) mainly due to the logarithmic calendar degradation (in a year or so), followed by a slow degradation stage (point B to point C) which is basically the stage the main lifetime of the battery depends on. This continues up to 80% capacity which is the defined threshold by OEMs for their EOL. After this point, battery degradation happens very fast (point C to point D) meaning that the number of deliverable cycles after 80% capacity is relatively small.

Modelling the SOH of retired EVBs uses kilometres travelled $VKT$, age of disposal, fuel efficiency $FE$, daily trip distance $DTD$, and battery size $BS$ to estimate the number of cycles that EVs travel as abstracted in Eq. 2 adopted from the previous study in which this formulae is represented in page 4 of [9]. Furthermore, this information is inserted in a battery estimation model to estimate the SOH of batteries at the time of vehicle disposal. Accordingly, the SOH of EVBs at EOL is a function of the total number of cycles $N$ delivered in vehicle lifetime, EOL age of vehicle, and the daily depth of discharges $DOD$ as Eq. 3 shows. The number of cycles $N$ in Eq. 2 is estimated based on average parameters extracted from real datasets as explained in page 4 of [9]. The VKT is averaged by extracting the recorded mileage of vehicles reaching EOL in the MOT dataset (Department-of-Transport, n.d.) [7] for different age of disposal. The DTD is adopted from a survey (National Transport Authority, 2018) [19] modelling the daily travel behaviour of drivers in Ireland. FE is obtained from [25] which follow the WLTP driving profile. However, this averaged number of full cycles could be spent differently based on driving behaviour in terms of charging and discharging and result in different SOH since discharging behaviour or $DOD$ is an effective factor in battery health estimation. This impact has been counted by a distribution of $DOD$ as expressed in Eq. 3 and Eq. 4, meaning that health of batteries is distributed across a range of $DODs$. Further details of this modelling, formulas and parameters can be found in page 4 of [9].

2 $$\begin{array}{c}N=f(VKT,age,FE,DTD,BS)\\\left\{\begin{array}{c}N:number\;of\;cycles\\VKT:vehicle\;kilometer\;travelled\\age:age\;of\;disposal\\FE:fuel\;efficiency\\DTD:daily\;distance\;travelled\\BS:battery\;size\end{array}\right.\end{array}$$

3 $$\begin{array}{c}{SOH}_{EOL}=SOH\left(N,age,DOD\sim dist(DOD)\right)\\\left\{\begin{array}{c}SOH_{EOL}:SOHatend\_of\_life\\age:age\;of\;disposal\\N:number\;of\;cylces\\dist\left(DOD\right):distribution\;of\;depth\;of\;discharge\end{array}\right.\end{array}$$

4 $$\begin{array}{c}{SOH}_{EOL}\sim dis\left({SOH}_{EOL}\right)=SOH\left(N,age,DOD\right)\sim dist(DOD)\\\left\{\begin{array}{c}SOH_{EOL}:SOHatend\_of\_life\\dist\left(SOH_{EOL}\right):distriubution\;of\;SOH_{EOL}\\N:number\;of\;cylces\\age:age\;of\;disposal\\dist\left(DOD\right):distribution\;of\;depth\;of\;discharge\end{array}\right.\end{array}$$

Moreover, since $N$ in Eq. 4 is a unique constant number for every age of disposal, the SOH is determinable for every age with a distribution over a range of $DOD$. Thus, the number of EOL EVB units with a specified SOH and age of disposal can be captured as Eq. 5 suggests. This equation shows that multiplying the number of units in a specific age category by the probability of a specific SOH to happen results in age and SOH identification of units. An excel file for this calculation is provided in the supplementary document.

5 $$\begin{array}{c}{EOL\_EVB}_{SOH,age}={PDF\left(SOH\right)}_{age}\ast{EOL\_EVB}_{age}\\\left\{\begin{array}{c}{EOL\_EVB}_{SOH,age}:number\;of\;end\_of\_life\;EV\;batteries\;for\;evergy\;age\;and\;SOH\\PDF:Probability\;distribution\;function\\age:age\;of\;disposal\end{array}\right.\end{array}$$

This above analysis so far enables us to classify EOL EVBs based on age and SOH. Accordingly, three reuse scenarios are extracted; In these scenarios, the SOH threshold for being qualified for a second life is set at above 80%, 85%, and 90% each, whereas below 80% is considered obsolete units. Similarly, to calculate the number of EOL EVBs above a SOH limit as a reuse threshold in every age category, Eq. 6 is applied wherein the likelihood of the SOH of batteries to be above the limit (cumulative distribution function) in a specific age category is multiplied to the number of units within that age category.

6 $$\begin{array}{c}{EOL\_EVB}_{SOH\_limit,age}={CDF\left({SOH}_{limit}\right)}_{age}\ast{EOL\_EVB}_{age}\\\left\{\begin{array}{c}\\{EOL\_EVB}_{SOH\_limit,age}:number\;of\;EOLEV\;batteries\;above\;a\;SOH\;threshold\;for\;evergy\;age\\CDF:Cumulative\;distribution\;function\\{CDF\left({SOH}_{limit}\right)}_{age}:CDF\;of\;SOH\;above\;the\;threshold\;limit\;for\;every\;age\end{array}\right.\end{array}$$

Capturing the repurposing capacity, their ultimate returning flow to the waste stream for potential recycling also needs to be approximated. For this, the second-life survival rate of these batteries needs to be estimated. The second-life survival rate of the retired EVBs again depends on their SOH and age at the time of disposal, thus is not a unique distribution for every EOL EVB. To model the survival distributions, the remaining calendar life of every category with a specified SOH and age is estimated as the top of the spectrum in second-life survival. This is achievable by finding the calendar life associated with each remained SOH. Technically retired EVBs can survive for the remaining calendar life in backup applications such as reserve services in the electricity market. However, they might go through earlier failures or be repurposed in more energy-intensive applications such as hybrid reserve and arbitrage trading in the electricity markets- as discussed in result section in page 6–8 of [9]- with a shorter lifetime. Thus, a peak at half of the calendar life is assumed for their distributions. Half of the calendar life is a rational assumption as batteries will typically be used for several arbitrage trading during a year for spiked prices, causing their lifetime to be shortened by half in a moderate case. Although they are best suited for reserve application, such markets will be overwhelmed specifically because the growth of the automobile battery market is far more rapid than stationary storage.

The second survival distribution curves are illustrated in the results section.

Having the number of units for reusable batteries and their second-life survival rate, their return to the waste flow is projected as the following formulae suggest. This calculation is available in the supplementary data.

7 $$\begin{array}{c}DLY{D\_EOL\_battery}_{age}={EOL\_EVB}_{SOH,age}\ast{2nd\_L}_{SOH,age}\\\left\{\begin{array}{c}DLY{D\_EOL\_battery}_{age}:delayed\;EOL\;batteries\;for\;every\;age\\{EOL\_EVB}_{SOH,age}:EOL\;batteries\;for\;every\;SOH\;and\;age\;of\;disposal\\{2nd\_L}_{SOH,age}:second\;life\;survival\;rate\;for\;every\;age\;and\;SOH\end{array}\right.\end{array}$$

The results are discussed in the following section.

## Results

**Fig. 3:**
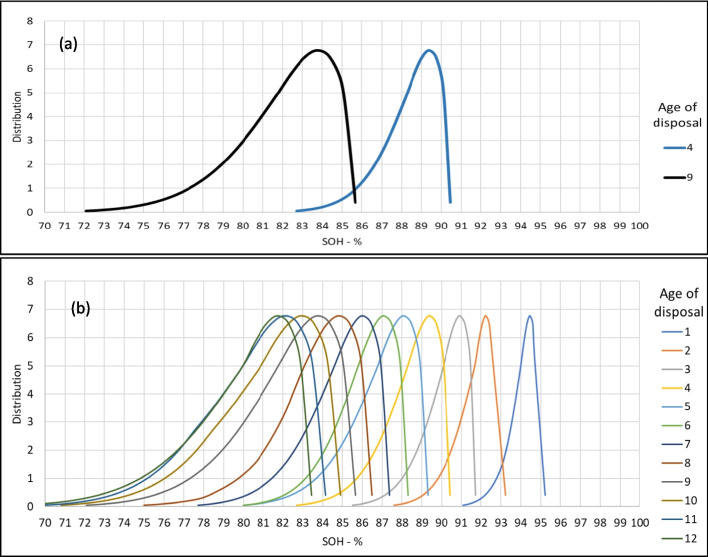
(**a** & **b**) Distributions of SOH of retired EVBs with various age of disposal

Results of SOH estimation for different age categorizations of EOL EVBs are shown in Fig. 3. Various ranges of SOH are expected for every age category with a peak of approximately 89% and 83% SOH for vehicles disposed at four and nine-year-old, as these averages were also represented in page 5 of [9]. Moreover, Fig. 2(b) shows the distribution of SOH for various ages of disposal up until the peak age of 12 years old. Since the ones after the age of 12 years old are likely to have below 80% remaining capacity on average, they are not counted in the SOH representation for repurposing but rather have been directly classified for recycling.

**Fig. 4:**
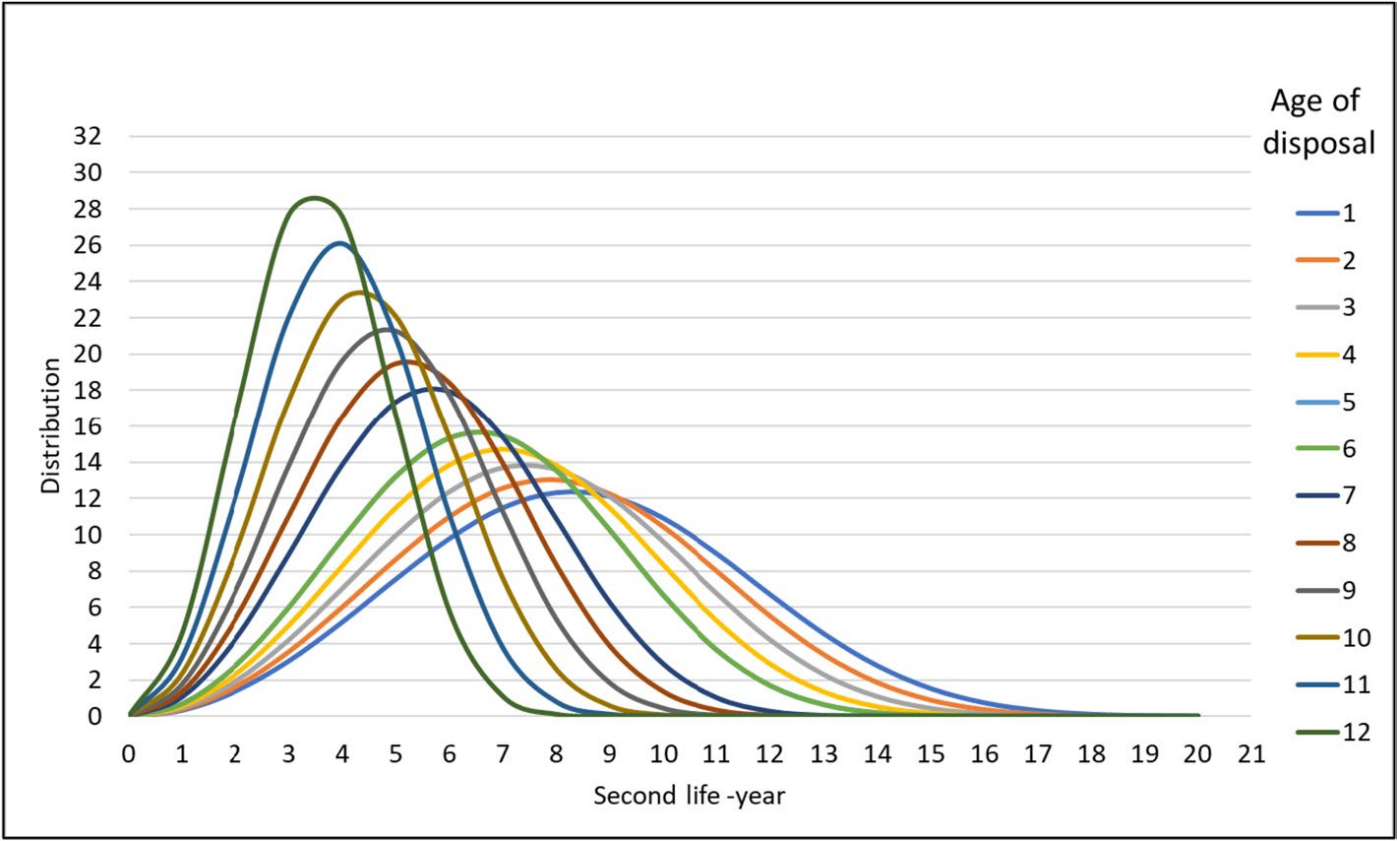
Distribution of second-life survival for retired EVBs with different age of disposal

Furthermore, Fig. 4 indicates a series of distribution curves for the potential second life of EVBs disposed of at different ages. According to these curves, a range of average second survival life of 4 to 9.5 years is expected for 12 to 1-year-old disposed of EVBs. However, the second-life survival rate is not taking the whole spectrum of SOH at the ages of disposal but is limited to the ones above 80% in every age category. For instance, the 12-year-old EVBs whose SOH is above 80% are likely to survive for four years at peak. Obviously, this is not covering the whole category of 12-year-olds as the SOH distributions in the previous figure show.

**Fig. 5:**
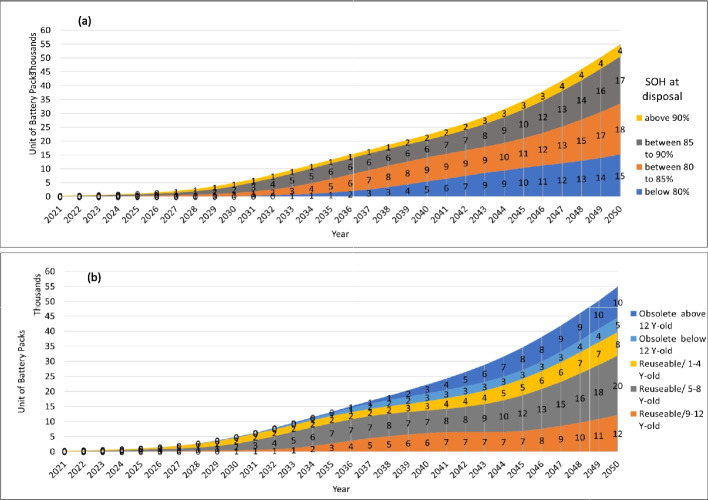
Results of the estimated units of EOL EVB packs classified by (**a**) SOH at disposal and (**b**) age of disposal

Having the distributions of SOH of EVBs at end of their first life and the second life survival rate of the ones with above 80% SOH, a hierarchical material flow analysis is conducted to project the reuse and recycling potential. Results are shown in Fig. 5, wherein Fig. 5(a) shows the SOH classification of EOL EVBs within the four groups of above 90%, between 85–90%, between 80–85%, and below 80% of SOH. The numbers show the units of EOL EVB packs within each hierarchy. As it can be observed, the 80–85% remained capacity units are the biggest portion of the waste with 18 thousand in 2050, and the second is 85–90% with 17 thousand. Whereas the above 90% units are four thousand units in 2050, comparatively a smaller portion, and the rest with below 80% capacity are 15 thousand in 2050. Looking at the percentage categorization in the figures in appendix-A, in the early waste flow, up to 2030 approximately, most of the units are above 80% healthy, wherein the share of above 90% is considerable due to the aggregation of EVBs coming from early failure vehicles, although the absolute values are not significant in early years. The share of the below 80% is increasing more rapidly for the last decade.

Moreover, Fig. 5(b) shows the age categorization for the same waste stream. Three age categories of 1–4, 5–8, and 9–12 are specified for reusable batteries i.e., above 80% SOH, whereas the rest would go to either above 12-year-old or below 12-year-old but obsolete (below 80% SOH) categories. Note that reusable age categorization in this work only considers the EOL EVBs below 12 years old because above this age threshold, the likelihood of a battery being healthy (above 80%) is quite low. According to these results, in 2050, the highest portion belongs to the age category of 5–8 with 20 thousand units, and the second one is 9–12 with 12 thousand units. The 1–4-year-old category contains 8 thousand units, whereas the obsolete ones below 12-year-old age are around five thousand units. And, finally, the above 12-year-old category contains 10 thousand of units of obsolete batteries. Almost the same pattern is observed for the percentage share of age categorization as the SOH categorization shown in Appendix-A, with an early start of younger batteries and overtaking of mature ones for the last decade.

**Fig. 6:**
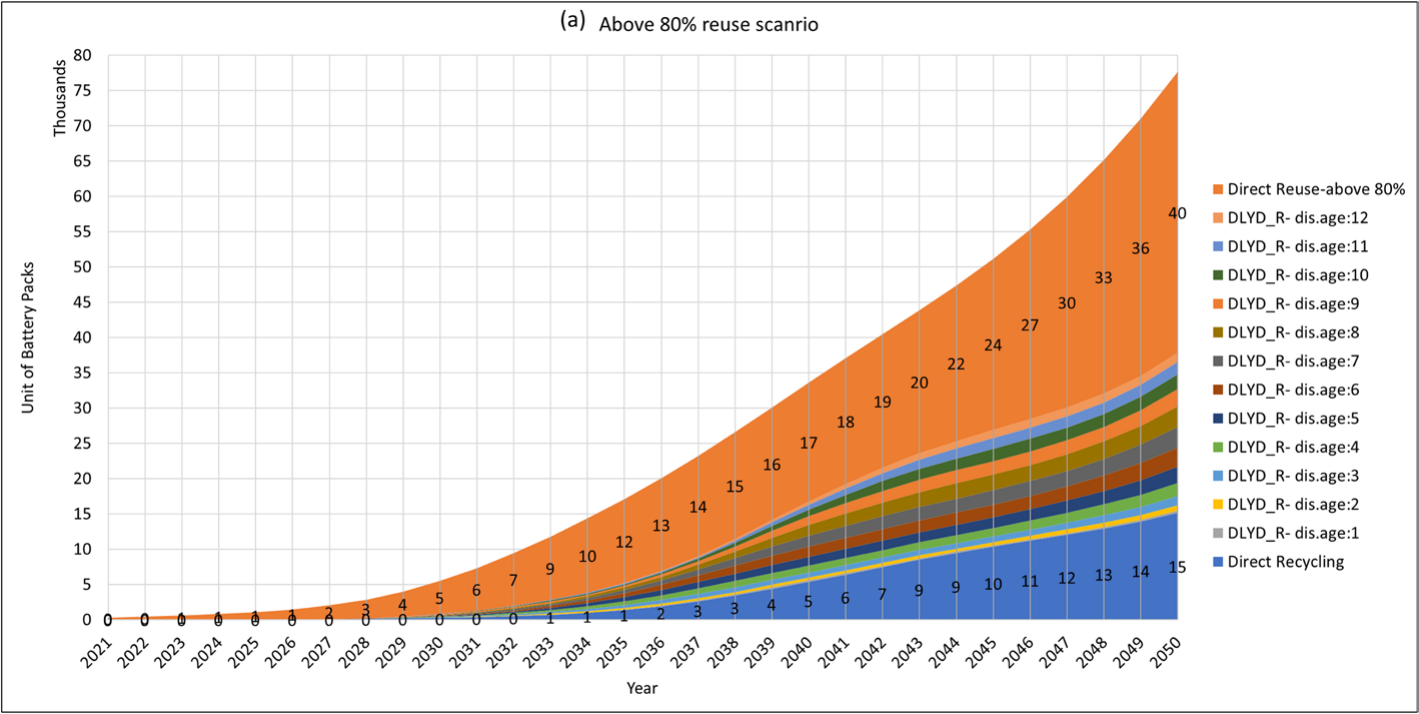

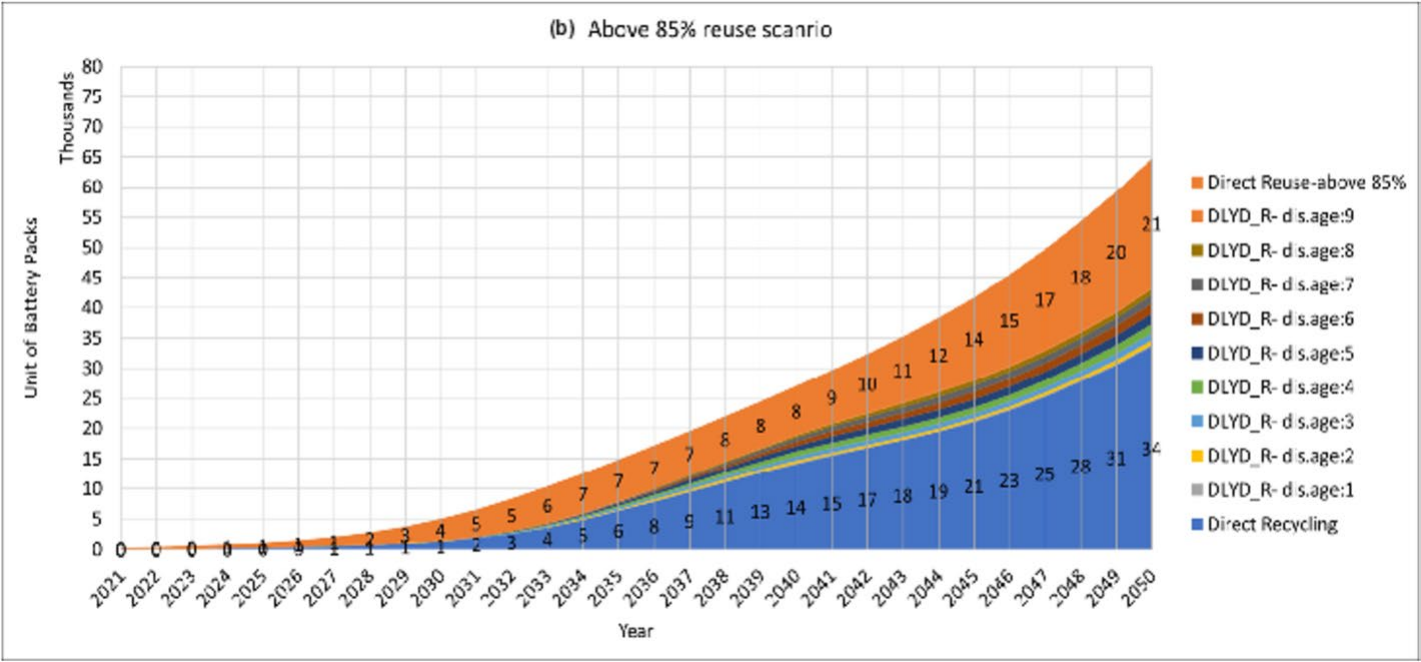

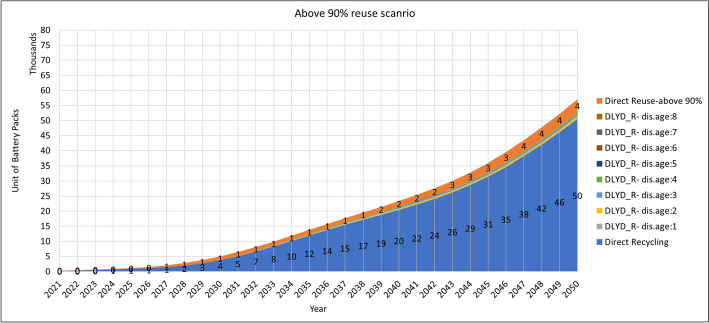
Estimated volumes of reuse, direct recycling, and delayed recycling in the (**a**) above 80% (**b**) above 85% and (**c**) above 90% reuse scenarios

Further in this study, we evaluated the effect of three reuse scenarios on the waste stream. In these scenarios, three levels of reuse threshold are considered in which batteries above 90%, 85%, and 80% will be reused. Results are shown in Fig. 6. As these curves show, reusing retired EVBs with above 80% SOH will end up having 38 thousand obsolete batteries in the year 2050, whereas 43 and 52 thousand obsolete units are expected in 2050 in above 85% and 90% reuse scenarios, respectively. Comparing these numbers with the no-reuse scenario at all, as in Fig. 5, the amount of obsolete EVB waste will decrease to 69%, 78%, and 94% in 2050. However, the numbers of obsolete EVB waste in Fig. 5 assume that the delayed obsolete battery waste is coming from the local repurposing applications, whereas this is not the current practice with electronic waste. Thus, this exclusion- even partly—will decrease the recycling capacity in the country. This issue needs attention both from the business development and the regulatory sides. Accordingly, the numbers of reusable units are estimated to be 40, 21, and 4 thousand units in reuse scenarios above 80%, 85%, and 90%, respectively. Of course, the above 80% repurposing is favourable for lifetime extension of the batteries as long as there are local repurposing business bodies to take care of them and adequate demand exists for second-life applications on the market.

## Conclusion

Projecting the recycling and repurposing perspective in the battery waste stream is a complicated task and incorporates technical and financial considerations. Although estimating this potential will help businesses to properly size and be created. Results in this study confirm a considerable repurposing potential in the battery waste stream. This work proposes a hierarchical representation of battery waste by their age and state of health, and further introduces three repurposing scenarios based on the expectation of the level of health of batteries at the time of disposal. This SOH categorization is useful for the businesses to know how much secondary supply of batteries they can access for high energy demanded applications for which the healthier batteries fit the best (above 85% or 90%), whereas the others (between 80 to 85%) can be used for less demanded back up storages. The possible second-use market alignment on the state of health hierarchy is that early repurposing businesses take place around the top shelf healthy batteries whereas a ‘wider health range’ repurposing happens when there is enough demand on the second application market.

,On the recycling side, the above 80% repurposing scenario shows a 50% rate of repurposing is likely in the year 2050, which leaves the other 50% for recycling, although this is the optimistic percentage since the experience from WEEE tells us that repurposing does not always take place in the original market; this is specifically important for the collection target, recycling facilities and needs consideration in ultimate EOL treatment in lower-income countries. The missing information on EOL vehicles already exists in the country and will likely continue with EVs as well [8, 24].

Higher recycling potential is estimated at almost 70% and more than 90% in above 85 and 90% repurposing scenarios, respectively. Whereas all are subject to the risk of being exported.

This projection also highlights that policy should seek to capture the reuse value of these vehicles as there is a danger that recyclers will only seek to recover material value. More importantly, a repurposing policy can help increase the resilience of the battery supply in case of material interruptions.

### Electronic supplementary material

Below is the link to the electronic supplementary material.

Supplementary file1 (XLSX 141 KB)

Supplementary file2 (XLSX 89 KB)

## Figures and Tables

**Fig. 7:**
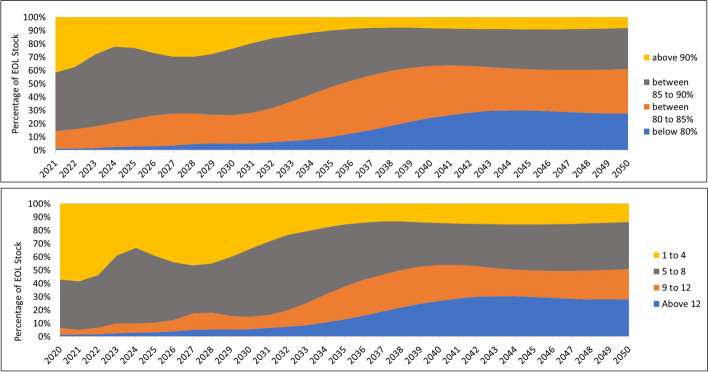
Estimated percentage of the units of EOL EVB packs classified by SOH at disposal and age of disposal

## Data Availability

The data that support the findings of this study is provide in the supplementary document.

## Appendix A

Figure 7
